# The National Pension Fund

**Published:** 1887-07-30

**Authors:** 


### THE NATIONAL PENSION FUND.

SINCE the publication of the former list of Institutions there has been a rapid increase in the number of names sent in. A new list will be published at the end of next month, and all those who have decided in their own minds that the Fund ought to be established, are requested to send in their names within the next few days.

---

MRS. S. P., Rugby.—Full particulars will be made known when the requisite number of names—2,000—shall have been sent in. In the meantime the pages of THE HOSPITAL will contain information from time to time. No responsibility is incurred by sending in names. Those doing so merely express their desire to join the Pension Fund if they approve the conditions when published.

---

MISS ROSS, Superintendent of Nurses at the Royal Infirmary, Glasgow, has read with very great interest the papers which have appeared in the THE HOSPITAL on the National Pension Fund, and will willingly add her name to the list. It is a much needed Fund, and every nurse ought to join. Many of the nurses will do so from this hospital, but they wish to hear more about it first.

From a Hospital Worker.

I should like my name to be added to those willing to co-operate in the National Pension Fund for Nurses. I have been in one of our large provincial hospitals for five years as probationer, and nurse, and then a sister of a large ward. I can therefore speak from experience, and consider that such a Fund would be most valuable both in cases where nurses have to retire permanently, or where in any case of illness from over-work they have to take a longer holiday than the institution to which they belong could allow them. Could it not be suggested that we subscribe one shilling a quarter to such a fund? I am taking needed rest at present, therefore I have no hospital address.

